# Sex-specific pain management and outcomes in pediatric elective posterior spinal fusion: a database review

**DOI:** 10.1007/s43390-026-01306-0

**Published:** 2026-02-21

**Authors:** Elizabeth Chan, Mason Dermott, Abigail Heims, Christina Colon-Sanchez, Devika A. Shenoy, Evan Schrader, Bradley Q. Fox, Ian Halliday, Stephanie Hendren, Anthony A. Catanzano

**Affiliations:** 1 School of Osteopathic Medicine, Campbell University, Buies Creek, NC USA; 2 Department of Orthopaedic Surgery, Duke University School of Medicine, Durham, NC USA

**Keywords:** Spinal fusion, Adolescent idiopathic scoliosis, Sex differences, Pain management

## Abstract

**Purpose:**

While sex-based differences in pain perception have been increasingly recognized, their reporting in adolescent patients undergoing spinal fusion (SF) remains inconsistent. This study examined the prevalence of sex-stratified outcomes in pain reporting following SF for adolescent idiopathic scoliosis (AIS).

**Methods:**

A systematic database review was conducted in accordance with a pre-registered protocol to identify studies (January 2015–January 2025) evaluating pain management in pediatric posterior spinal fusion. Eligible studies reported sex-stratified outcomes related to pain scores, reporting frequency, or management modalities. Screening and data extraction were performed independently by two reviewers, with adjudication by a third.

**Results:**

Of 207 studies undergoing full text review, 43 (21%) met inclusion criteria, representing 27,691 patients (29.4% male; 70.6% female; mean age 15 years). Twenty studies disaggregated pain scores by sex, 23 reported sex-specific pain management, and only two reported both. Several studies identified greater pain burden and chronic postoperative pain in females, whereas others found no sex differences. Some studies reported higher opioid or benzodiazepine use among males, while others demonstrated prolonged postoperative opioid use in females. Eleven studies reported no differences. Heterogeneity in pain assessment tools and analgesic regimens limited comparability.

**Conclusions:**

Sex-stratified outcomes remain under-reported and inconsistently analyzed in AIS patients undergoing SF. Only 20% of studies provided sex-specific outcomes, and fewer reported both pain score and pain management data. Standardized reporting using uniform pain metrics and stratified analyses is needed to clarify differential pain trajectories and guide equitable, individualized perioperative care.

**Supplementary Information:**

The online version contains supplementary material available at 10.1007/s43390-026-01306-0.

## Introduction

Posterior spinal fusion (PSF) remains the gold standard in the treatment of severe adolescent idiopathic scoliosis (AIS) and is often associated with significant postoperative pain. Effective pain management is a vital component of a positive postoperative experience and recovery, promoting less psychological stress and a quicker return to daily activities. To optimize pain management and avoid adverse effects, a multimodal approach is utilized, including pharmacologic, physical, and psychological methods [1]. Achieving successful pain management accelerates recovery and enables patients to reach their postoperative milestones.

However, pain management is not a one-size-fits-all approach and varies among patients, considering differences in pain perception, response to treatment, and prescribing patterns of providers [2–4]. This is especially true when comparing male and female patients. Females have been found to have a higher prevalence of pain and an increased burden of musculoskeletal complaints—such as osteoarthritis, chronic widespread pain, and back or neck pain—compared to males [5–9]. While this has initially been explored in adult populations, in children and adolescents, there is less consensus on the pain experience between males and females [10–12]. Having a better understanding of individual patient factors can affect how providers decide what modalities may be most effective, as well as provide more accurate expectations for pharmacological pain management. While the exact mechanisms underlying these differences remain unclear, research suggests that variations in pain experiences are attributed to differences in sex-specific hormone production levels, anatomy, and inflammatory responses [13, 14]. The recognition of these differences has increased, especially in examining how implicit biases in healthcare affect clinical decision-making and patient outcomes [15, 16]. Moreover, recognizing these sex-based differences is clinically relevant as postoperative analgesic decisions directly influence recovery trajectories, opioid exposure, and risk of postsurgical chronic pain. In the absence of sex-stratified reporting, providers may rely on uniform pain management protocols that inadequately address individual patient needs in addition to inadvertently reinforcing the existing treatment biases. As enhanced recovery protocols and multimodal analgesic pathways become more standardized, identifying whether these approaches perform equally across sexes is necessary to ensure safe, equitable, and individualized perioperative management.

Interestingly, while pain management after PSF for AIS has been extensively studied, stratifying pain management outcomes by patient sex is not routinely performed. Evaluating the reporting of sex-specific use of pain management modalities and their effects on orthopaedic procedure outcomes can highlight the insufficient consideration given by providers when prescribing certain pharmacological and non-pharmacological pain management modalities. Thus, this study aims to analyze current literature and quantify the reporting of sex-specific differences in pain management and pain outcomes in adolescent patients undergoing elective PSF for AIS. We hypothesize that a minority of studies will include sex stratification within their analysis of pain scores and pain management.

## Methods

### Study design and literature search

This article adhered to database review guidelines outlined by Kwon et al. and followed the Preferred Reporting Items for Systematic Reviews and Meta-Analyses (PRISMA) criteria [17]. A medical librarian with expertise in systematic searching comprehensively conducted a search on April 28, 2025 in Medline (via Ovid) for studies published from January 1, 2015, to April 28, 2025. The search utilized appropriate controlled vocabulary and keywords related to spinal fusion, pain, and pediatrics (Supplement 1). All search results were imported into Covidence (Veritas Health Innovation, Melbourne, Australia) for duplication removal, screening, and data extraction [18].

### Inclusion criteria

Inclusion criteria consisted of pediatric patients (< 18 years of age) who had undergone PSF, with sex included as an exposure variable, and pain-related outcomes of interest (pain score values, frequency of pain reporting, pain management modalities—both pharmacological and non-pharmacological—and time to pain management). Studies were excluded if they were editorials, conference abstracts, case reports, meta-analyses, commentaries, in vitro or animal studies, did not include sex-specific analysis, non-English language, or were published prior to 2015.

### Screening

An initial abstract screening was performed in which two authors independently voted on each abstract, and if disagreements arose, a third author resolved the issue [19]. Following abstract screening, the availability of the full manuscript was confirmed, and articles selected for full-text review were assessed. A complete review of each text utilizing the inclusion and exclusion criteria mentioned above was conducted, and additionally, including the papers in which sex was differentiated in the results and excluding those in which sex was only mentioned as a description of demographics. As with the abstract screening, two authors voted on each article, and a third reviewer would resolve any disagreements.

### Data extraction

From each article, the following was collected: DOI, title, lead author, country of study origin, aim of the study, dates the study took place, study design, data source, type of surgery, disease the surgery was for, total number of patients, age of total patients, number of male/female patients, age of male/female patients, additional demographics of male/female patients, pain score data stratified by sex, and pain management data stratified by sex. For this review, *pain scores* refer to measures of patients’ subjective pain perception utilizing standardized measures such as the visual analogue scale (VAS), numerical rating scale (NRS), or disease-specific questionnaires, such as the Scoliosis Research Society (SRS-22/24) and the Pain Catastrophizing Scale for Children (PCS-C). *Pain management* encompasses pharmacological, non-pharmacological, or multimodal strategies to reduce postoperative pain and improve outcomes. Data were extracted independently by two authors, with consensus by a third, and analyzed using descriptive statistics in Microsoft Excel.

## Results

### Study selection

**Fig. 1:**
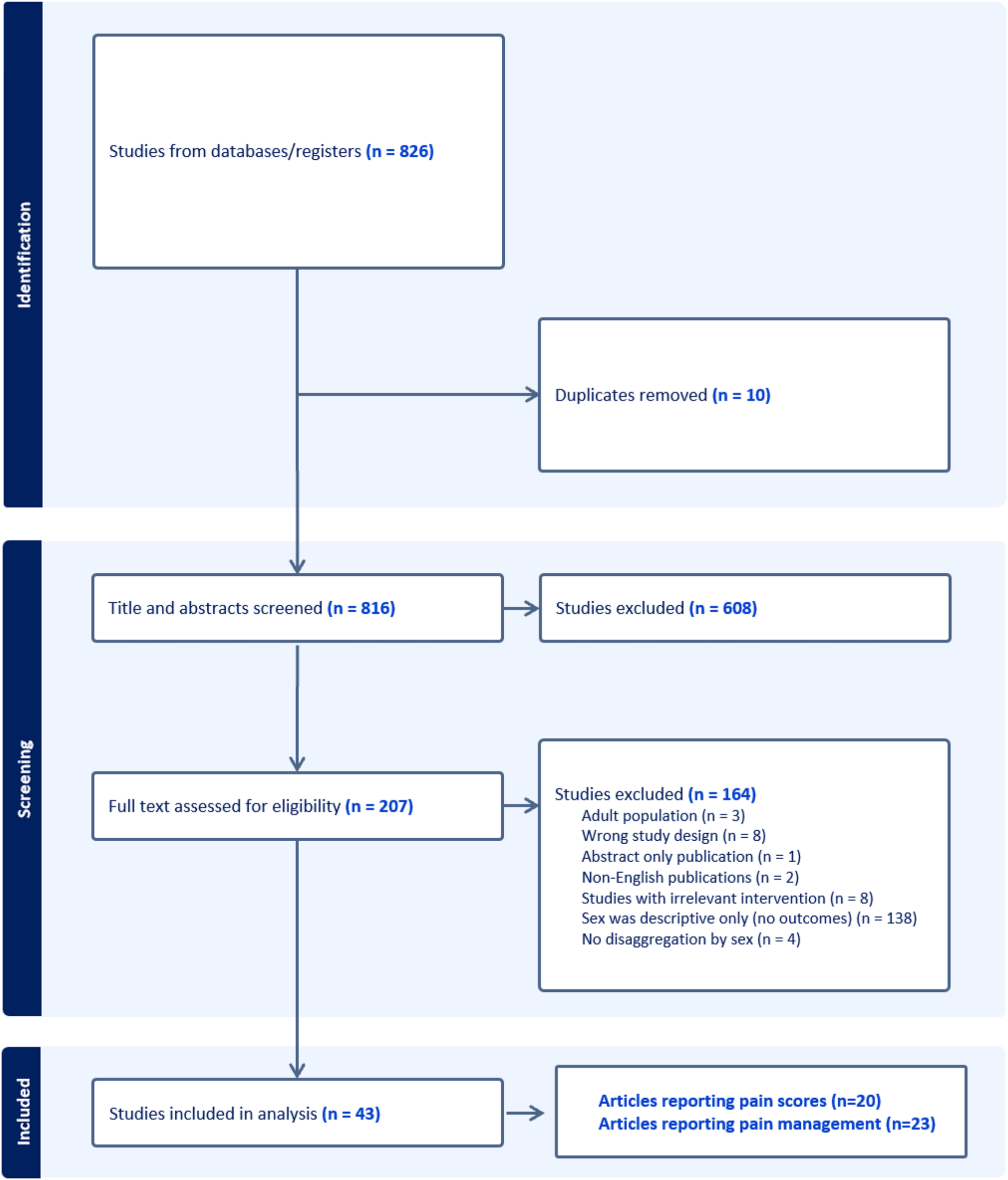
Prisma flow diagram for studies with sex-specific data stratification

The initial database search identified 826 studies, of which 10 duplicates were removed. After screening titles and abstracts, a total of 207 studies published from January 2015 to January 2025 were eligible for full-text review. After full-text review of these studies, 43 studies (21%) met the inclusion criteria and were included in the analysis (Fig. 1).

### Study characteristics

The analyzed studies included a total of 27,691 patients, of which 8148 (29.4%) were males and 19,547 (70.6%) were females. The mean age of all patients was 15.0 years (range: 1–21 years), with females (mean 14.7 years) slightly younger than males (mean 15.7 years). The vast majority of surgeries were correction of AIS, with only four articles, including other surgeries. Over 80% of the cases in each of the four articles pertained to AIS, with the remaining surgeries for pectus excavatum, spondylolisthesis, or discectomy. Thirty-three (76.7%) of the studies were conducted in the United States, with other articles published in the United Kingdom (1), Poland (2), Canada (2), Finland (1), France (1), China (1), Malaysia (1), and Japan (1). Study designs consisted of cohort, case–control, and randomized clinical trials. Detailed study characteristics are included in Table 1.

**Table 1:** Characteristics of studies with sex-specific data stratification

Study Title	Author	Year	Total number of patients	Age of total patients Mean ± SD (Range)	Males N	Females N
The Role of Cytokines in Acute and Chronic Postsurgical Pain in Pediatric Patients after Major Musculoskeletal Surgeries	Chidambaran et al	2024	112	15.3 (13.5–17)	51	61
Predictors of Extended Length of Hospital Stay in Adolescent Idiopathic Scoliosis Patients Undergoing Posterior Segmental Instrumented Fusion: An Analysis of 407 Surgeries Performed at a Large Academic Center	Sultan et al	2019	407	(10–25)	85	322
Factors associated with increased back pain in primary thoracic adolescent idiopathic scoliosis 10 years after surgery	Bastrom et al	2022	171	10.6 ± 0.8	30	141
Pain Catastrophizing Throughout the Perioperative Period in Adolescents With Idiopathic Scoliosis	Chabot et al	2021	83	15.66 (10–19)	26	57
Predicting the pain continuum after adolescent idiopathic scoliosis surgery: A prospective cohort study	Chidambaran et al	2017	144	14.44 ± 1.97	38	106
Association of OPRM1 A118G variant with risk of morphine-induced respiratory depression following spine fusion in adolescents	Chidambaran et al	2015	88	14.59 ± 1.89 (10–18.9)	29	59
Systems Biology Guided Gene Enrichment Approaches Improve Prediction of Chronic Post-surgical Pain After Spine Fusion	Chidambaran et al	2021	171	14.48 (10–18)	42	129
Preoperative SRS pain score is the primary predictor of postoperative pain after surgery for adolescent idiopathic scoliosis: an observational retrospective study of pain outcomes from a registry of 1744 patients with a mean followâ€ ‘up of 3.4 years	Hwang et al	2020	1744	(10–18)	356	1388
Quality of life in children and adolescents undergoing spinal deformity surgery	McKean et al	2017	545	15.14 ± 2.07 (10–18)	120	425
Association of CYP2D6 genotype predicted phenotypes with oxycodone requirements and side effects in children undergoing surgery	Merchant et al	2022	193	15.9 ± 0.25	138	55
Factors leading to postoperative pain in adolescent idiopathic scoliosis patients including sagittal alignment and lumbar disc degeneration	Mimura et al	2019	101	14.8 ± 2.3	8	93
Pediatric Pain Screening Tool: A Simple 9-Item Questionnaire Predicts Functional and Chronic Postsurgical Pain Outcomes After Major Musculoskeletal Surgeries	Narayanasamy et al	2022	109	14.67 (13.24–15.95)	35	74
Prospective 10-year follow-up assessment of spinal fusions for thoracic AIS: radiographic and clinical outcomes	Newton et al	2020	174	14.5 ± 2.1	30	144
HRQoL assessment by SRS-30 for Chinese patients with surgery for Adolescent Idiopathic Scoliosis (AIS)	Ng et al	2015	104	16.25	20	84
The Potential Role of Preoperative Pain, Catastrophizing, and Differential Gene Expression on Pain Outcomes after Pediatric Spinal Fusion	Perry et al	2021	36	14 + -1.75 (10–17)	9	27
Pain Catastrophizing Influences Preoperative and Postoperative Patient-Reported Outcomes in Adolescent Idiopathic Scoliosis	Ramo et al	2022	189	14.7 ± 1.81	35	154
Pilot Investigation of Somatosensory Functioning and Pain Catastrophizing in Pediatric Spinal Fusion Surgery	Sieberg et al	2023	32	13.9 ± 1.7 (10–17)	7	25
A cluster of high psychological and somatic symptoms in children with idiopathic scoliosis predicts persistent pain and analgesic use 1 year after spine fusion	Voepel-Lewis et al	2018	95	(10–17)	23	72
A High Psychological and Somatic Symptom Profile and Family Health Factors Predict New or Persistent Pain During Early Adolescence	Voepel-Lewis et al	2017	49	(10–17)	13	36
Predicting Acute Postoperative Pain Trajectories and Long-Term Outcomes of Adolescents after Spinal Fusion Surgery	Ocay et al	2020	106	15.4 ± 2.0	25	81
Rapid Recovery Pathway for Postoperative Treatment of Adolescent Idiopathic Scoliosis	Ahdoot et al	2021	44	(11–21)	8	36
Multimodal pain control in adolescent posterior spinal fusion patients: a double-blind, randomized controlled trial to validate the effect of gabapentin on postoperative pain control, opioid use, and patient satisfaction	Anderson et al	2020	50	14.6	12	38
Clinical and Economic Outcomes Associated With Use of Liposomal Bupivacaine Versus Standard of Care for Management of Postsurgical Pain in Pediatric Patients Undergoing Spine Surgery	Ballock et al	2021	10,189	(1–17)	LB = 138; Non-LB = 4019	LB = 235; Non-LB = 5797
Minimalistic approach to enhanced recovery after pediatric scoliosis surgery	Barnett et al	2023	92	(10–18)	SR *p* = 10; ER *p* = 14	SR *p* = 34; ER *p* = 34
Pain Medication Use Two Years After Adolescent Idiopathic Scoliosis Fusion Surgery	Bastrom et al	2024	2595	14.7 ± 2.9	494	2101
Lidocaine as an element of multimodal analgesic therapy in major spine surgical procedures in children: a prospective, randomized, double-blind study	Batko et al	2020	41	13 (8–15)	17	24
The Role of Liposomal Bupivacaine in Multimodal Pain Management Following Posterior Spinal Fusion for Adolescent Idiopathic Scoliosis	Changoor et al	2024	119	14.5 ± 2.3	23	96
Perioperative methadone for posterior spinal fusion in adolescents: Results from a double-blind randomized-controlled trial	Fons et al	2024	47	(10–18)	9	38
Are We Prescribing Our Patients Too Much Pain Medication? Best Predictors of Narcotic Usage After Spinal Surgery for Scoliosis	Grant et al	2016	61	14.9	19	42
Association between intraoperative remifentanil use and postoperative hyperalgesia in adolescent idiopathic scoliosis surgery: a retrospective study	Hasan et al	2023	97	14 ± 2 (10–18)	19	78
A Retrospective Comparison of Intrathecal Morphine and Epidural Hydromorphone for Analgesia Following Posterior Spinal Fusion in Adolescents with Idiopathic Scoliosis	Hong et al	2017	40	(10–20)	4	36
Effects of Opioid-Reduced Anesthesia During Scoliosis Surgery in Children: A Prospective Observational Study	Julien-Marsollier et al	2021	69	< 18	13	56
Fentanyl versus remifentanil-based TIVA for pediatric scoliosis repair: does it matter?	Kars et al	2019	62	(11–20)	13	49
Intravenous, Perioperatively Administered Lidocaine Regulates Serum Pain Modulators Concentrations in Children Undergoing Spinal Surgery	Koscielniak-Merak et al	2020	44	12	20	24
The Association Between Adjuvant Pain Medication Use and Outcomes Following Pediatric Spinal Fusion	Rosenberg et al	2017	7349	(10–18)	1682	5667
High inpatientâ€ ‘opioid consumption predicts extended length of hospital stay in patients undergoing spinal fusion for adolescent idiopathic scoliosis	Sandhu et al	2025	1042	14 (11–17)	239	803
The Impact of Standardized Recovery Pathways on Language Barriers and Inpatient Pain Management	Segal et al	2023	160	(10–20)	47	113
Preoperative Patient Education and Smaller Prescription Quantity Reduce Opioid Use After Posterior Spinal Fusion for Adolescent Idiopathic Scoliosis: Results of a Prospective Study	Yang et al	2022	49	14.1 ± 1.8	13	36
Risk Factors for Prolonged Postoperative Opioid Use After Spinal Fusion for Adolescent Idiopathic Scoliosis	Yang et al	2019	511	(10–19)	124	387
Rapid recovery pathway without epidural catheter analgesia for surgical treatment of adolescent idiopathic scoliosis: a comparative study	Colon et al	2023	53	(11–19)	13	40
Evaluation of Gabapentin and Clonidine Use in Children Following Spinal Fusion Surgery for Idiopathic Scoliosis: A Retrospective Review	Choudhry et al	2019	127	(11–20)	26	101
Sex related difference in postoperative pain and opioid use following posterior spinal fusion for adolescent idiopathic scoliosis	Collis et al	2024	137	(10–17)	30	107
Pregabalin and Persistent Postoperative Pain Following Posterior Spinal Fusion in Children and Adolescents: A Randomized Clinical Trial	Helenius et al	2021	64	10–21)	22	42

Of the 43 included articles, 20 stratified pain scores by sex, and 23 stratified pain management strategy outcomes by sex. Two studies included both pain score and pain management data stratified by sex and were included in both analyses [22, 23]. Sex-stratified pain scores were reported for the following pain scales: numerical rating scale, SRS-22, VAS, and the PCS-C. Sex-stratified pain management modalities included: recovery pathways (involve replacing narcotic medications with alternative analgesics, such as acetaminophen, gabapentinoids, NSAIDs, and diazepam, among others), opioid-based regimens (e.g., morphine, methadone, and patient-controlled analgesia), opioid-sparing medications (e.g., gabapentinoids, liposomal bupivacaine, ketorolac, and lidocaine), adjuvants (dexmedetomidine, ketamine, and benzodiazepines), and multimodal analgesia combinations.

### Reporting of non-numeric pain assessments

Twelve studies reported pain outcomes by sex via demographic measures and grouped patients into cohorts, such as symptom clusters, pain trajectories, or psychological measures as opposed to numerical values. Female patients were more likely to report chronic postsurgical pain (CPSP) (OR = 6.756, *p* < 0.001) as reported by Narayanasamy et al. Utilizing area under the curve analysis, Merchant et al. found that females had increased postoperative pain burden. Voepel-Lewis et al. found that compared to males, females were significantly less likely to be in the *No Pain/High Psychological and Somatic Symptoms* group (OR 0.72, 95% CI 0.63–0.82, *p* < 0.001) and more likely to be in the Pain/Low Psychological and Somatic Symptoms group (OR 1.26, 95% CI 1.15–1.38, *p* < 0.001), with no significant difference in the Pain/High Psychological and Somatic Symptoms group (OR 0.96, 95% CI 0.84–1.09, *p* = 0.502) [24–26]. Furthermore, Sieberg et al. observed that female patients more frequently reported moderate-to-high pain catastrophizing scores both pre- and postoperatively and Chabot et al. noted that females were predominantly represented in the highest PCS-C—a measurement of exaggerated negative thoughts and feelings related to pain—during the perioperative period [27, 28].

However, not all studies found a link between pain and sex, as several studies found no statistical significance regarding differences in pain scores between sexes [26, 28–32]. Three additional studies did not report *p* values. Moreover, Hwang et al. assessed males and females in postoperative “pain” and “no pain” groups and found significantly more females in the no pain versus pain group [33]. However, the authors did not directly compare males to females in the study’s analysis. Full descriptions of studies reporting pain scores are reported in Table 2.

**Table 2:** Non-numeric pain assessments

Title	Author	Pain scale	Male patients	Female patients	Findings
Pediatric Pain Screening Tool: A Simple 9-Item Questionnaire Predicts Functional and Chronic Postsurgical Pain Outcomes After Major Musculoskeletal Surgeries	Narayanasamy, Suryakumar et al	Patients with no chronic postsurgical pain as measured with a numerical rating scale of < 4 over the previous month or during the time of pain assessment at 6 months post-surgery or 12 months after surgery	31	39	Female sex was associated with higher odds for CPSP (6.756 (95% CI 2.165–21.090; *p* < 0.001) in both cohorts
		Patients with chronic postsurgical pain as measured with a numerical rating scale of ≥ 4 over the previous month or during the time of pain assessment at 6 months post-surgery or 12 months after surgery	4	34	
A High Psychological and Somatic Symptom Profile and Family Health Factors Predict New or Persistent Pain During Early Adolescence	Voepel-Lewis, Terri et al	No pain (numerical rating scale) + low psychological and somatic symptoms	2923	2652	Compared to males, females were significantly less likely to be in the No Pain/High Psychological and Somatic Symptoms group (OR 0.72, 95% CI 0.63–0.82, *p* < 0.001) and more likely to be in the Pain/Low Psychological and Somatic Symptoms group (OR 1.26, 95% CI 1.15–1.38, *p* < 0.001), with no significant difference in the Pain/High Psychological and Somatic Symptoms group (OR 0.96, 95% CI 0.84–1.09, *p* = 0.502)
		No pain (numerical rating scale) + high psychological and somatic symptoms	752	503	
		Pain (numerical rating scale) + low psychological and somatic symptoms	1537	1709	
		Pain (numerical rating scale) + high psychological and somatic symptoms	833	710	
Preoperative SRS pain score is the primary predictor of postoperative pain after surgery for adolescent idiopathic scoliosis: an observational retrospective study of pain outcomes from a registry of 1744 patients with a mean follow-up of 3.4 years	Hwang, Steven et al	No postoperative pain group	323	1206	There were significantly more females in the no pain group compared to the pain group (*p* = 0.049) In the subgroup analysis of Lenke 1 and 2 curve patterns, a significantly higher proportion of females were in the no pain group compared to the pain group (85% vs. 78%, *p* = 0.044)
		Postoperative pain group: Pain as Complication or SRS 22 ≤ 3	33	182	
		Subgroup analysis of Lenke 1 and 2 curve patterns; no pain	222	787	
		Subgroup analysis of Lenke 1 and 2 curve patterns: pain	45	134	
The Potential Role of Preoperative Pain, Catastrophizing, and Differential Gene Expression on Pain Outcomes after Pediatric Spinal Fusion	Perry, Mallory et al	Visual analogue scale to report non-persistent post-surgical pain	7	16	Sex was not associated with persistent pain outcomes (*p* = 0.391)
		Visual analogue scale to report persistent post-surgical pain	2	11	
Factors associated with increased back pain in primary thoracic adolescent idiopathic scoliosis 10 years after surgery	Bastrom, Tracey P. et al	Scoliosis Research Society pain scores below normal (≤ 2 standard deviations below average for controls of similar age/sex from published literature)	23%	11%	There was no statistically significant difference between males and females in the number of reporting below-normal pain scores (*p* = 0.08)
		Scoliosis Research Society pain scores within/above the control range	76%	89%	
Factors leading to postoperative pain in adolescent idiopathic scoliosis patients including sagittal alignment and lumbar disc degeneration	Mimura, Tetsuhiko et al	Scoliosis Research Society			There was no association between sex and preoperative SRS-22r pain score (*p* = 0.91) or 2-year postoperative SRS-22r pain score (*p* = 0.91)
Pain Catastrophizing Throughout the Perioperative Period in Adolescents With Idiopathic Scoliosis	Chabot, Bianca et al	PCS-C: Low (0–14)	8	14	There is no statistically significant difference in sex distribution between the pain catastrophizing groups (*p* = 0.103)
		PCS-C: Medium Pain (15–25)	14	21	
		PCS-C: High Pain (26–43)	4	22	
Predicting the pain continuum after adolescent idiopathic scoliosis surgery: A prospective cohort study	Chidambaran, V et al	Pain in males compared to females using the numerical rating scale			There was no difference between males and females for acute pain (*p* = 0.181), chronic pain (*p* = 0.83, or persistent pain (*p* = 0.73)
A cluster of high psychological and somatic symptoms in children with idiopathic scoliosis predicts persistent pain and analgesic use 1 year after spine fusion	Voepel-Lewis, Terri et al	High symptom cluster, which is characterized by elevated scores across multiple dimensions (depression, fatigue, and numerical pain rating scales)	4	24	There is no statistical difference between the low and high symptom clusters in terms of female sex distribution (OR 0.42, 95% CI 0.13–1.38, *p* = 0.192) There is no statistical difference in long-term pain interference scores between males and females (β = –0.53, 95% CI –3.73 to 2.67, *p* = 0.741)
		Low symptom cluster	19	48	
Predicting Acute Postoperative Pain Trajectories and Long-Term Outcomes of Adolescents after Spinal Fusion Surgery	Ocay, Don Daniel et al	Pain trajectory 1 = who reported mild pain that remained constant throughout the acute postoperative period	8	18	
		Trajectory 2 = patients who reported mild and mild-to-moderate pain that remained relatively constant throughout the acute postoperative period	7	22	
		Trajectory 3 = mild-to-moderate pain immediately following surgery, but increased steadily to moderate pain by postoperative day five	8	18	
		Trajectory 4 = patients who reported moderate pain immediately following surgery that remained relatively constant throughout the acute postoperative period	2	23	
Pilot Investigation of Somatosensory Functioning and Pain Catastrophizing in Pediatric Spinal Fusion Surgery	Sieberg, Christine et al	Catastrophizing Scale- Children (PCS-C): Preoperative Low Pain (1–3)	4	10	
		PCS-C: Preoperative Moderate Pain (4–6)	3	7	
		PCS-C: Preoperative High Pain (7–10)	0	6	
		PCS-C: Postoperative Low Pain (1–3)	4	12	
		PCS-C: Postoperative Moderate Pain (4–6)	1	6	
		PCS-C: Postoperative High Pain (7–10)	0	4	
Pain Catastrophizing Influences Preoperative and Postoperative Patient-Reported Outcomes in Adolescent Idiopathic Scoliosis	Ramo, Brandon et al	Elevated pain catastrophizing scale score ≥ 30	3	17	17 females and 3 males reported clinically elevated PCS scores (PCS ≥ 30, corresponding to the ≥ 75th percentile)
		Pain catastrophizing scale score < 30	32	137	

### Reporting of numerical pain scores

Nine out of twenty articles that stratified pain score data by sex compared numerical pain scores (Table 3). Newton et al. found significant pain score differences between PSF patients when compared to “normal” individuals (male, *p* = 0.004; female, *p* < 0.001). However, there was no further analysis of this outcome while controlling for sex. Helenius et al. found that females had higher SRS-24 pain scores (*p* = 0.014) and Collis et al. reported that females were found to have higher pain scores using VAS on post-operative day 0 (*p* < 0.0001) and day 1 (*p* = 0.03), as well as at the first physical therapy evaluation (*p* < 0.001) [22, 23]. On the second physical therapy evaluation, however, the difference between sexes was determined to be insignificant (*p* = 0.37). Other studies reported no statistically significant differences in pain scores between males and females [34–36]. McKean et al. found no significant difference in preoperative SRS-22 scores (*p* = 0.753) between the sexes, although males did have statistically significantly better pain scores than females at 2 years postoperatively (*p* < 0.0001) [37]. Sieburg et al. found that females demonstrated significantly higher pre-operative pain scores (using a numerical rating scale) (*p* = 0.02); however, there were no differences between pain postoperatively [27]. This study also found no difference between sexes for average PCS-C, mechanical detection thresholds, mechanical pain thresholds, and pressure pain thresholds (*p* > 0.05). Merchant et al. concluded that the median area under the curve for female pain scores was significantly higher than that of the males (*p* < 0.001) [25].

**Table 3:** Numerical pain scores

Title	Author	Pain scale	Males	Females	Findings
Quality of life in children and adolescents undergoing spinal deformity surgery	McKean, Greg et al	SRS-22: preop	3.64 ± 0.66	3.61 ± 0.66	Males and females had similar pre-operative total SRS-22 scores (*p* = 0.753). Males had significantly better pain scores compared to females at 2-year postoperative (*p* < 0.0001)
		SRS-22: 2 years postop	4.77 ± 0.32	4.54 ± 0.60	
Pregabalin and Persistent Postoperative Pain Following Posterior Spinal Fusion in Children and Adolescents: A Randomized Clinical Trial	Helenius, Linda et al	Scoliosis Research Society			Female patients reported significantly greater pain than male patients (*p* = 0.014)
Systems Biology Guided Gene Enrichment Approaches Improve Prediction of Chronic Post-surgical Pain After Spine Fusion	Chidambaran, Vidya et al. (2021)	numerical rating scale at 6–12 months postop	0	2	There was no significant difference between male and female pain scores (*p* = 0.331)
Association of OPRM1 A118G variant with risk of morphine-induced respiratory depression following spine fusion in adolescents	Chidambaran, Vidya et al. (2015)	numerical rating scale	4.5	4.6	Results of regression with pain scores as outcome female vs male: β = 0.11 ± 0.21, *p* = 0.59 postoperative pain scores (numerical rating scale): *p*-value females vs males = 0.63
Prospective 10-year follow-up assessment of spinal fusions for thoracic AIS: radiographic and clinical outcomes	Newton, Peter et al	SRS-22: Patients with surgery for scoliosis	4.6	4.4	Both the male and female patients had more symptomatic pain compared to sex-matched ‘normal’ individuals at 10-year follow-up (M: *p* = 0.004; F: *p* < 0.001). Compared with those that had spinal deformities and remained untreated, both males and females who were surgically treated had better outcomes in all domains (*p* = 0.01)
		SRS-22: Patients without scoliosis	4.2	4.1	
Sex related difference in postoperative pain and opioid use following posterior spinal fusion for adolescent idiopathic scoliosis	Collis, Reid W. et al	VAS Postoperative Day 0	3.6 ± 0.5	5.0 ± 1.8	Females had a higher VAS score on Postoperative Day 0 (*p* < 0.0001), Postoperative Day 1 (*p* = 0.03), and physical therapy evaluation 1 (*p* < 0.001). There was no significant difference between sexes on physical therapy evaluation 2 (*p* = 0.37)
		VAS Postoperative Day 1	4.0 ± 0.6	4.9 ± 1.9	
Systems Biology Guided Gene Enrichment Approaches Improve Prediction of Chronic Post-surgical Pain After Spine Fusion	Chidambaran, Vidya et al	VAS at first physical therapy evaluation	3.8 ± 0.6	5.3 ± 2	There was no significant difference between male and female pain scores (*p* = 0.331)
Association of OPRM1 A118G variant with risk of morphine-induced respiratory depression following spine fusion in adolescents	Chidambaran, Vidya et al	VAS at second physical therapy evaluation	4.4 ± 0.9	4.9 ± 2.3	There was no significant difference in mean postoperative pain scores between males and females in the unadjusted comparison (*p* = 0.63). After adjusting for other variables, the beta was 0.11 ± 0.21, *p* = 0.59, and thus the difference between females and males was still not significant
Association of CYP2D6 genotype predicted phenotypes with oxycodone requirements and side effects in children undergoing surgery	Merchant, Soroush et al	Median (interquartile range) of the area under the curve for numerical pain scores during the immediate postoperative period	71.1 (41.15–115.3)	111.22 (73.89–137.28)	Median of area under the curve for pain scores was higher for females compared to males (*p* < 0.001)
Pilot Investigation of Somatosensory Functioning and Pain Catastrophizing in Pediatric Spinal Fusion Surgery	Sieberg, Christine et al	Self-reported pain score (numerical rating scale): preoperative	0.9 ± 1.2 (range: 0 to 3)	2.4 ± 2.5 (range: 0 to 8	Females demonstrated significantly higher preoperative pain scores (*p* = 0.02), and the postoperative score differences were insignificant
		Self-reported pain score (numerical rating scale): 4–6 months postoperative	0.9 ± 1.6 (range: 0 to 4)	2.0 ± 2.1 (range: 0 to 7)	
		Preoperative/ postoperative: Average PCS-C	11/7.2	16.61/14.55	No significant difference between sexes in Average PCS-C scores (*p* > 0.05)
		Mechanical detection thresholds: preoperative at non-pain site (grams)	2.58	2.8	There were no significant correlations between males and females for mechanical detection thresholds, mechanical pain thresholds, and pressure pain thresholds (*p* > 0.05)
		Mechanical detection thresholds: preoperative at pain site (grams)	2.86	3.05	
		Mechanical detection thresholds: postoperative at non-pain site (grams)	2.83	2.97	
		Mechanical detection thresholds: postoperative at pain site (grams)	3.48	3.26	
		Mechanical pain thresholds: preoperative at non-pain site (grams)	4.41	4.95	
		Mechanical pain thresholds: preoperative at pain site (grams)	4.65	5.07	
		Mechanical pain thresholds: postoperative at non-pain site (grams)	4.62	5.18	
		Mechanical pain thresholds: postoperative at pain site (grams)	5.20	5.21	
		Pressure pain thresholds: preoperative at non-pain site (grams)	33.92	20.01	
		Pressure pain thresholds: preoperative at pain site (grams)	25.09	16.20	
		Pressure pain thresholds: postoperative non-pain site (grams)	42.16	30.69	
		Pressure pain thresholds: postoperative pain site (grams)	46.42	22.36	
HRQoL assessment by SRS-30 for Chinese patients with surgery for Adolescent Idiopathic Scoliosis (AIS)	Kin Wah Ng, Bobby et al	SRS-22: preoperative pain score	4.31 ± 0.46	4.21 ± 0.59	No significant difference between male and female scores (preoperative pain scores: *p* = 0.459; discharge pain score: *p* = 0.499; follow-up pain scores: *p* = 0.524)
		SRS-22: discharge pain score	2.05 ± 1.79	2.33 ± 1.65	
		SRS-22: follow-up pain score	2.40 ± 2.26	2.06 ± 2.11	

### Reporting of pain management strategies

Twenty-three studies included pain management results, strategy, or modality stratified by sex (Table 4). Several studies found that males have higher opioid and benzodiazepine usage. For example, Grant et al. found higher opioid use among males (*p* = 0.002), and Rosenberg et al. indicated an association between males prescribed benzodiazepines and use of additional pain medications following pediatric spinal surgery (*p* = 0.05) [38, 39]. In addition, Segal et al. (2023) stated that females had a decreased probability of receiving additional forms of patient-controlled analgesia (PCA) (*p* = 0.008) [40]. In contrast, Yang et al. (2019) reported that prolonged (> 6 weeks) opioid use following surgery was correlated with female sex (*p* < 0.05) [41]. Collis et al. reported that more morphine milligram equivalents (MME) were given to females both immediately following PSF (24 h postoperative) as well as throughout their entire hospitalization (*p* = 0.013 and *p* = 0.048, respectively) [22]. However, this study did not find any significant differences between the amount of intraoperative medications given to males and females.

**Table 4:** Pain management strategies

Article title	Author	Pain management	Males N (%)	Females N (%)	Findings
Are We Prescribing Our Patients Too Much Pain Medication? Best Predictors of Narcotic Usage After Spinal Surgery for Scoliosis	Grant, Daniel et al	Narcotics: low pill use	2 (0.03)	15 (25)	Male sex was associated with higher narcotic use (*p* = 0.002)
		Narcotics: average pill use	7 (11)	22 (36)	
		Narcotics: high pill use	10 (16)	5 (0.08)	
		Received Refills	Yes: 8, No: 11	Yes: 9, No: 33	Sex-based analysis on refills was not discussed
The Association Between Adjuvant Pain Medication Use and Outcomes Following Pediatric Spinal Fusion	Rosenberg, Rebecca et al	Ketorolac	980 (13)	336 (0.05)	Males have greater adjuvant pain modalities with benzodiazepine prescription (*p* = 0.05)
		GABA	373 (0.05)	1264 (17)	
		Benzodiazapine	1682 (23)	5667 (77)	
The Impact of Standardized Recovery Pathways on Language Barriers and Inpatient Pain Management	Segal, Kathryn et al	Patient-controlled analgesia	47 (29)	113 (71)	Females had lower odds of receiving additional forms of patient-controlled analgesia (*p* = 0.008)
Risk Factors for Prolonged Postoperative Opioid Use After Spinal Fusion for Adolescent Idiopathic Scoliosis	Yang, Scott et al	Opioid use < 6 weeks	116 (23)	345 (68)	Female sex was associated with longer post-operative opioid use (*p* < 0.05)
		Opioid use > 6 weeks	6 (0.01)	42 (0.08)	
Clinical and Economic Outcomes Associated With Use of Liposomal Bupivacaine Versus Standard of Care for Management of Postsurgical Pain in Pediatric Patients Undergoing Spine Surgery	Ballock, Robert et al	Liposomal bupivacaine	138 (0.01)	235 (0.02)	Group sex distribution was insignificant (*p* = 0.128)
		Non-liposomal bupivacaine	4019 (39)	5797 (57)	
Minimalistic approach to enhanced recovery after pediatric scoliosis surgery	Barnett, Scott et al	Standard recovery protocol	10 (11)	34 (37)	Group sex distribution was insignificant (*p* = 0.482)
		Enhanced recovery protocol	14 (15)	34 (37)	
Lidocaine as an element of multimodal analgesic therapy in major spine surgical procedures in children: a prospective, randomized, double-blind study	Batko, Ilona et al	Lidocaine	9 (22)	13 (32)	Group sex distribution was insignificant (*p* = 0.938)
		Control	8 (20)	11 (27)	
The Role of Liposomal Bupivacaine in Multimodal Pain Management Following Posterior Spinal Fusion for Adolescent Idiopathic Scoliosis	Changoor, Stuart et al	PRN Meds: acetaminophen, oxycodone, and IV morphine + liposomal bupivacaine	10 (0.08)	43 (36)	Group sex distribution was insignificant (*p* = 0.909)
		PRN Meds: acetaminophen, oxycodone, and IV morphine, no liposomal bupivacaine	13 (11)	53 (45)	
Perioperative methadone for posterior spinal fusion in adolescents: Results from a double-blind randomized-controlled trial	Fons, Roger et al	methadone	4 (0.09)	2 (0.04)	Group sex distribution was insignificant (*p* = 0.08)
		morphine	5 (11)	17 (36)	
Association between intraoperative remifentanil use and postoperative hyperalgesia in adolescent idiopathic scoliosis surgery: a retrospective study	Hasan, M. Shahnaz et al	Low-dose Remifentanil	11 (11)	30 (31)	Group sex distribution was insignificant (*p* = 0.124)
		High-dose Remifentanil	8 (0.08)	48 (49)	
Effects of Opioid-Reduced Anesthesia During Scoliosis Surgery in Children: A Prospective Observational Study	Julien-Marsollier, Florence et al	Opioid-based anesthesia (propofol, sufentanil, atracurium)	5 (0.07)	31 (45)	Group sex distribution was insignificant (*p* = 0.27)
Intravenous, Perioperatively Administered Lidocaine Regulates Serum Pain Modulators Concentrations in Children Undergoing Spinal Surgery	Koscielniak-Merak, Barbara et al	Lidocaine	10 (43)	13 (56)	There was no difference in morphine requirements between sexes in the lidocaine group (*p* = 0.477) or the control group (*p* = 0.78)
		Control	10 (48)	11 (52)	
High inpatient opioid consumption predicts extended length of hospital stay in patients undergoing spinal fusion for adolescent idiopathic scoliosis	Ratnesh S. Sandhu, Mani et al	Low Milligram Morphine Equivalents (MME)	70 (0.07)	190 (18)	Group sex distribution was insignificant (*p* = 0.141)
		Medium MME	118 (11)	405 (39)	
		High MME	51 (0.05)	208 (20)	
Preoperative Patient Education and Smaller Prescription Quantity Reduce Opioid Use After Posterior Spinal Fusion for Adolescent Idiopathic Scoliosis: Results of a Prospective Study	Yang, Daniel et al	Low opioid use	4 (0.08)	8 (16)	Group sex distribution was insignificant (*p* = 0.899)
		Average opioid use	7 (14)	22 (45)	
		High opioid use	2 (0.04)	4 (0.08)	
Evaluation of Gabapentin and Clonidine Use in Children Following Spinal Fusion Surgery for Idiopathic Scoliosis: A Retrospective Review	Choudhry, Dinesh K. et al	morphine: patient-controlled analgesia	6 (0.05)	36 (28)	Group sex distribution was insignificant (*p* = 0.096)
		morphine: patient-controlled analgesia + Gabapentin	14 (11)	31 (24)	
		morphine: patient-controlled analgesia + Gabapentin + Clonidine	6 (5)	34 (27)	
Sex related difference in postoperative pain and opioid use following posterior spinal fusion for adolescent idiopathic scoliosis	Collis, Reid W. et al	Intraoperative Acetaminophen use (mg)	16 males (53%); 440.2 ± 429.1	68 females (64%) 523.5 ± 416.1	There were no differences between males and females in the amounts given of any intraoperative medications (*p* > 0.05)
		Intraoperative Bupivacaine (Non-liposomal) use (mg)	16 patients (53%) 80.8 ± 78.7	43 patients (40%) 102.6 ± 76	
		Intraoperative Bupivacaine (Liposomal) use (mg)	16 patients (53%) 137.4 ± 132.9	72 patients (67%) 172.5 ± 125.3	
		Intraoperative Lidocaine use (mg)	23 patients (77%) 5.7 ± 3.4	99 patients (93%) 6.7 ± 2.6	
		Intraoperative Dexmedetomidine use (mcg)	5 patients (17%) 3.1 ± 7.2	9 patients (8%) 1.7 ± 6.2	
		Intraoperative Diazepam use (mg)	1 patient (3%) 0.2 ± 0.9	3 patients (3%) 0.1 ± 0.6	
		Intraoperative Fentanyl use (mcg)	30 patients (100%) 340.7 ± 180.4	102 patients (95%) 321.0 ± 186.8	
		Intraoperative Hydromorphone use (mg)	19 patients (63%) 0.4 ± 0.5	74 patients (69%) 0.5 ± 0.5	
		Intraoperative Ketamine use (mg)	14 patients (47%) 20.7 ± 26.1	67 patients (63%) 29.8 ± 25.1	
		Intraoperative Ketorolac use (mg)	30 patients (100%) 15.3 ± 13.5	73 patients (68%) 17.1 ± 12.5	
		Intraoperative Lidocaine use (mg)	18 patients (60%) 40.3 ± 40.4	70 patients (65%) 38.4 ± 32.8	
		Intraoperative Methocarbamol use (mg)	9 patients (0.3%) 224.2 ± 361.7	40 patients (37%) 299.7 ± 404.7	
		Intraoperative Morphine use (mg)	1 patient (0.3%) 0.3 ± 1.5	1 patient (.9%) 0.04 ± 0.4	
		Intraoperative Remifentanil use (mcg)	3 patients (.3%) 208.3 ± 676.2	8 patients (7%) 140.0 ± 537.9	
		First 24 h morphine milligram equivalents (mg)	31.5 ± 17.6	42.2 ± 21.5	Females received more morphine milligram equivalents 24-h postoperatively (*p* = 0.013)
		Total postoperative morphine milligram equivalents (mg)	51.3 ± 29.4	63.9 ± 30.6	Females had higher total postoperative morphine milligram equivalents during hospitalization (*p* = 0.048)
Rapid Recovery Pathway for Postoperative Treatment of Adolescent Idiopathic Scoliosis	Ahdoot, Eli S. et al	Rapid recovery pathway	13 (30)	19 ( 43)	Pain management outcomes analyzed by treatment groups only
		Conventional recovery pathway	5 (11)	17 (39)	
Multimodal pain control in adolescent posterior spinal fusion patients: a double-blind, randomized controlled trial to validate the effect of gabapentin on postoperative pain control, opioid use, and patient satisfaction	Anderson, Devon et al	Gabapentin	5 (10)	19 (38)	Pain management outcomes analyzed by treatment groups only
		Placebo	7 (14)	19 (38)	
Pain Medication Use Two Years After Adolescent Idiopathic Scoliosis Fusion Surgery	Bastrom, Tracey P. et al	Narcotics	11 (0.004)	29 (0.01)	Pain management outcomes analyzed by treatment groups only
		Non-narcotics	58 (0.02)	503 (19)	
		No medication	425 (16)	1569 (60)	
A Retrospective Comparison of Intrathecal Morphine and Epidural Hydromorphone for Analgesia Following Posterior Spinal Fusion in Adolescents with Idiopathic Scoliosis	Hong, Rebecca et al	Intrathecal Morphine	2 (0.05)	18 (45)	Pain management outcomes analyzed by treatment groups only
		Epidural Hydromorphone	2 (0.05)	18 (45)	
Fentanyl versus remifentanil-based TIVA for pediatric scoliosis repair: does it matter?	Kars, Michelle et al	Opioid-reducing anesthesia (-methyl-d-aspartate antagonists (ketamine), ± 2 agonists (dexmedetomidine, clonidine), anti-inflammatory drugs (NSAIDs), or regional/local anesthesia to avoid opioid administration during surgery)	8 (13)	25 (40)	Pain management outcomes analyzed by treatment groups only
Rapid recovery pathway without epidural catheter analgesia for surgical treatment of adolescent idiopathic scoliosis: a comparative study	Colon, Luis Felipe et al	Rapid recovery pathway	6 (11)	12 (23)	Pain management outcomes analyzed by treatment groups only
		Control	7 (13)	28 (53)	
Pregabalin and Persistent Postoperative Pain Following Posterior Spinal Fusion in Children and Adolescents: A Randomized Clinical Trial	Helenius, Linda et al	Pregabalin	12 (19)	21 (33)	Pain management outcomes analyzed by treatment groups only
		Placebo	10 (16)	21 (33)	

Eleven of the 23 studies found no significant differences in pain management strategies between males and females. No differences were observed with liposomal bupivacaine versus non-liposomal bupivacaine (Ballock et al.), standard versus enhanced recovery protocols (Barnett et al.), or lidocaine compared with control (Batko et al.; Koscielniak-Merak et al.). Similarly, as-needed medication use—including acetaminophen, oxycodone, and IV morphine—with and without liposomal bupivacaine showed no differences between sexes (Changoor et al.). Opioid-based medications, such as methadone versus morphine (Fons et al.), low- versus high-dose remifentanil (Hasan et al.), opioid-based anesthesia (Julien-Marsollier et al.), and MME dosing (Ratnesh et al.; Yang et al.), also revealed no significant differences [42–51]. Finally, regimens involving gabapentin and clonidine in addition to morphine-based patient-controlled analgesia demonstrated no sex-related differences Choudhry et al. [52]. While these studies reported pain management use in both males and females, other studies did not conduct further analysis to determine if there was a significant difference between sexes.

## Discussion

This database review highlights the significant under-reporting of sex-stratified outcomes in studies analyzing post-operative pain management in PSF for AIS. Despite the large body of literature on postoperative pain in AIS, only 21% of the studies in this review examined sex-based differences, 11% reported pain management strategies, and only 3.9% used numerical pain scores. Importantly, just two studies provided data on both outcomes (pain scores and pain management strategies and modalities), underscoring the inconsistency of how sex differences are addressed. Heterogeneity in measurement tools further complicates interpretation. Unlike the cervical and lumbar spine, which have NDI and ODI, respectively, no validated equivalent exists for the thoracic spine. Studies in this review employed a variety of instruments—including the numerical rating scale, SRS-22, SRS-24, pain catastrophizing scale for children, median area under the curve, and VAS. Standardizing pain reporting analogous to approaches used in other spinal regions, such as combining SRS-22 and VAS could aid in interpretability for this area.

Similarly, the breadth of analgesic strategies studied included opioid-based regimens, multimodal analgesia, and opioid-sparing approaches, reflecting the evolving landscape of perioperative pain management. This variability highlights the significance of our scoping review, emphasizing the individualized nature of pain management and underscoring the need for more detailed, sex-stratified reporting as well as standardized outcome measures to enable meaningful comparisons across studies.

Of the twenty studies that reported pain scores, only eight reported sex-stratified outcome measures. Among the studies that reported sex-specific outcomes in non-numeric pain scores, four studies categorized females in higher pain cohorts. Still, six studies found no significant difference between sexes. Three studies did not report *p* values, further highlighting inconsistent statistical reporting among studies. It is important to note that AIS surgery has a much higher prevalence among females (77%) than males (23%), which is consistent with the distribution of sexes among the studies reviewed [20, 21]. In the studies reviewed, the distribution of sexes in each pain management group was kept consistent with the average sex distribution of AIS to preserve group similarity. In studies using pain scale results to determine groups, the number of male and female patients comprising each group was included in the results of the studies.

For studies that reported pain scores as an outcome, four used SRS-22, one used VAS, one used the median of the area under the curve for numerical pain scores, and three used a numerical self-reported pain score. Such methodological variability precludes direct comparisons and reveals a broader lack of standardization in how pain is measured following PSF. In addition, the variety in scales posed a challenge in comparing the results of the studies throughout this review, and demonstrates the need for larger, more thorough studies on the management of pain and differences between sexes. How data were analyzed between sexes in each of these studies was also variable, with only two studies providing the statistical significance of different pain outcomes between the sexes, while the other six presented data comparing sex pain outcomes independently without directly stating the relationship between outcome and sex.

Regarding pain management strategies, the literature is similarly inconsistent. Some studies reported males receiving higher doses of opioids or benzodiazepines (Grant et al. and Rosenberg et al.), whereas others reported a pattern of prolonged postoperative opioid consumption in females Yang et al. [38, 39, 41] Across the 23 articles reporting sex-specific results in pain management data, a range of modalities was utilized. In addition to the variety of analgesics used, the dosages were also variable from study to study. Interestingly, only six studies (26%) stratified their analyses by sex, and none found significant differences.

From a clinical perspective, these gaps in reporting are significant and make interpretation difficult for clinicians. Evidence suggests that pain perception differs between sexes [9, 53]. For example, a widespread cortical nociceptive response is more likely to occur in females [25]. However, these differences are not just limited to the spine. A 2021 study of over 2000 pediatric participants identified the prevalence of persistent musculoskeletal pain greater among girls than boys, with the disparity widening over time [10]. Despite this, sex-stratified pain reporting remains inconsistent in pediatric orthopedic literature, including for post-PSF outcomes for AIS. Optimizing postoperative pain management is imperative for surgeons to enhance recovery outcomes, patient satisfaction, and decrease prolonged opioid use. Future literature should aim for more consistency in exposure and outcome measures and be more inclusive of patient factors, such as sex. Because the current literature largely fails to investigate sex differences in postoperative pain after spinal fusion, it risks perpetuating existing treatment biases that disproportionately affect females [7, 16, 54]. Addressing these gaps will require larger, methodologically consistent studies that stratify outcomes by sex and incorporate standardized pain metrics.

### Limitations

This study has several limitations. Although focused on AIS patients aged 10–18, 10 of the studies included participants older than 18, with one extending to age 25. In addition, four studies included a small subset of patients outside of the scope of this paper, including patients treated for pectus excavatum and spondylolisthesis. However, these studies were still included, because the patient subgroup comprised less than 20% of the study populations and outcomes were not stratified by this group. Furthermore, the variability in reporting of pain management modalities limited direct comparisons and hindered the development of standardized sex-specific recommendations. In addition, some studies published results with sex-stratified data without a statistical comparison between groups.

## Conclusion

This study evaluated the prevalence of sex-stratified pain reporting in the AIS literature. Future research should employ consistent outcome measures and incorporate sex stratification, as this is critical for optimizing individualized pain management strategies in PSF. Further investigations focusing on specific methods or targeted analgesic approaches may help yield more definitive conclusions. Ultimately, systematic incorporation of sex-stratified data across all age groups will be essential to improve equity, refine perioperative protocols, and guide evidence-based, patient-centered care.

## Supplementary Information

Below is the link to the electronic supplementary material.

Supplementary file1 (PDF 122 KB)

## Data Availability

This study is a database review of previously published literature. All data analyzed are available in the cited articles. The full list of included studies is provided in the reference list and/or tables. The search strategy can be found in the supplementary materials.

## References

[1] El-Tallawy SN, Nalamasu R, Salem GI, LeQuang JAK, Pergolizzi JV, Christo PJ (2021) Management of musculoskeletal pain: an update with emphasis on chronic musculoskeletal pain. Pain Ther 10(1):181–209 33575952 10.1007/s40122-021-00235-2 PMC8119532

[2] Segal NA, Nilges JM, Oo WM (2024) Sex differences in osteoarthritis prevalence, pain perception, physical function and therapeutics. Osteoarthritis Cartilage 32(9):1045–1053 38588890 10.1016/j.joca.2024.04.002

[3] Sodhi N, Qilleri A, Aprigliano C, Danoff JR (2025) One size does not fit all: women experience more pain than men after total knee arthroplasty. J Arthroplasty 40(4):880–886 39307204 10.1016/j.arth.2024.09.028

[4] Paller CJ, Campbell CM, Edwards RR, Dobs AS (2009) Sex-based differences in pain perception and treatment. Pain Med 10(2):289–299 19207233 10.1111/j.1526-4637.2008.00558.x PMC2745644

[5] Fillingim RB, King CD, Ribeiro-Dasilva MC, Rahim-Williams B, Riley JL (2009) Sex, gender, and pain: a review of recent clinical and experimental findings. J Pain 10(5):447–485 19411059 10.1016/j.jpain.2008.12.001 PMC2677686

[6] Gerdle B, Björk J, Cöster L, Henriksson K, Henriksson C, Bengtsson A (2008) Prevalence of widespread pain and associations with work status: a population study. BMC Musculoskelet Disord 9(1):102 18627605 10.1186/1471-2474-9-102 PMC2488345

[7] Templeton KJ (2020) Sex and gender issues in pain management. J Bone Jt Surg 102(Suppl 1):32–35 10.2106/JBJS.20.00237 32251123

[8] Ellermeier W, Westphal W (1995) Gender differences in pain ratings and pupil reactions to painful pressure stimuli. Pain 61(3):435–439 7478686 10.1016/0304-3959(94)00203-Q

[9] Mogil JS (2020) Qualitative sex differences in pain processing: emerging evidence of a biased literature. Nat Rev Neurosci 21(7):353–365 32440016 10.1038/s41583-020-0310-6

[10] Picavet HSJ, Gehring U, Van Haselen A, Koppelman GH, Van De Putte EM, Vader S et al (2021) A widening gap between boys and girls in musculoskeletal complaints, while growing up from age 11 to age 20 ‐ the PIAMA birth cohort study. Eur J Pain 25(4):902–912 33405263 10.1002/ejp.1719 PMC8048429

[11] Earp BD, Monrad JT, LaFrance M, Bargh JA, Cohen LL, Richeson JA (2019) Featured article: gender bias in pediatric pain assessment. J Pediatr Psychol 44(4):403–414 30615163 10.1093/jpepsy/jsy104

[12] Boerner KE, Birnie KA, Caes L, Schinkel M, Chambers CT (2014) Sex differences in experimental pain among healthy children: a systematic review and meta-analysis. Pain 155(5):983–993 24508752 10.1016/j.pain.2014.01.031

[13] Cairns BE (2007) The influence of gender and sex steroids on craniofacial nociception. Headache 47(2):319–324 17300382 10.1111/j.1526-4610.2006.00708.x

[14] Failla MD, Beach PA, Atalla S, Dietrich MS, Bruehl S, Cowan RL et al (2024) Gender differences in pain threshold, unpleasantness, and descending pain modulatory activation across the adult life span: a cross sectional study. J Pain 25(4):1059–1069 37956742 10.1016/j.jpain.2023.10.027 PMC10960699

[15] FitzGerald C, Hurst S (2017) Implicit bias in healthcare professionals: a systematic review. BMC Med Ethics 18(1):19 28249596 10.1186/s12910-017-0179-8 PMC5333436

[16] Guzikevits M, Gordon-Hecker T, Rekhtman D, Salameh S, Israel S, Shayo M et al (2024) Sex bias in pain management decisions. Proc Natl Acad Sci U S A 121(33):e2401331121 39102546 10.1073/pnas.2401331121 PMC11331074

[17] Kwon J, Pelletiers W, Galloway Peña J, Van Duin D, Ledbetter L, Baum K et al (2024) Participant diversity in United States randomized controlled trials of antibacterials for *Staphylococcus aureus* infections, 2000–2021. Clin Infect Dis 79(1):141–147 38306502 10.1093/cid/ciae049 PMC11259209

[18] Covidence systematic review software. Melbourne, Australia: Veritas Health Innovation

[19] Megafu M, Guerrero O, Yendluri A, Uwefoh M, Li X, Kocher MS et al (2025) The lack of reporting social determinants of health in pediatric orthopaedic randomized controlled trials. J Pediatr Orthop 45(1):22–27 39171369 10.1097/BPO.0000000000002801

[20] Konieczny MR, Senyurt H, Krauspe R (2013) Epidemiology of adolescent idiopathic scoliosis. J Child Orthop 7(1):3–9 24432052 10.1007/s11832-012-0457-4 PMC3566258

[21] Sung S, Chae HW, Lee HS, Kim S, Kwon JW, Lee SB et al (2021) Incidence and surgery rate of idiopathic scoliosis: a nationwide database study. Int J Environ Res Public Health 18(15):8152 34360445 10.3390/ijerph18158152 PMC8346015

[22] Collis RW, Dry T, Chan G, Lim P, Oswald T (2024) Sex related difference in postoperative pain and opioid use following posterior spinal fusion for adolescent idiopathic scoliosis. Spine Deform 12(3):711–715 38329603 10.1007/s43390-024-00826-x PMC11068828

[23] Helenius L, Yrjala T, Oksanen H, Pajulo O, Loyttyniemi E, Taittonen M et al (2021) Pregabalin and persistent postoperative pain following posterior spinal fusion in children and adolescents: a randomized clinical trial. J Bone Joint Surg Am. 10.2106/JBJS.21.00153 34424869 10.2106/JBJS.21.00153

[24] Narayanasamy S, Yang F, Ding L, Geisler K, Glynn S, Ganesh A et al (2022) Pediatric pain screening tool: a simple 9-item questionnaire predicts functional and chronic postsurgical pain outcomes after major musculoskeletal surgeries. J Pain 23(1):98–111 34280572 10.1016/j.jpain.2021.06.014 PMC8783955

[25] Merchant S, Prows CA, Yang F, Ding L, MacDonald J, Zhang X et al (2022) Association of CYP2D6 genotype predicted phenotypes with oxycodone requirements and side effects in children undergoing surgery. Ann Transl Med 10(23):1262 36618804 10.21037/atm-2022-58 PMC9816853

[26] Voepel-Lewis T, Caird MS, Tait AR, Farley FA, Li Y, Malviya S et al (2018) A cluster of high psychological and somatic symptoms in children with idiopathic scoliosis predicts persistent pain and analgesic use 1 year after spine fusion. Paediatr Anaesth 28(10):873–880 30302887 10.1111/pan.13467

[27] Sieberg CB, Lunde CE, Wong C, Manganella J, Starkweather AR, Sethna N et al (2023) Pilot investigation of somatosensory functioning and pain catastrophizing in pediatric spinal fusion surgery. Pain Manag Nurs 24(1):27–34 36564325 10.1016/j.pmn.2022.11.001 PMC9925410

[28] Chabot B, Sweatman H, Ocay DD, Premachandran S, Roy M, Ferland CE (2021) Pain catastrophizing throughout the perioperative period in adolescents with idiopathic scoliosis. Clin J Pain 37(9):688–697 34265790 10.1097/AJP.0000000000000962 PMC8360666

[29] Perry M, Sieberg CB, Young EE, Baumbauer K, Singh V, Wong C et al (2021) The potential role of preoperative pain, catastrophizing, and differential gene expression on pain outcomes after pediatric spinal fusion. Pain Manag Nurs Off J Am Soc Pain Manag Nurses 22(1):44–49 10.1016/j.pmn.2020.05.007 PMC8742610 32771349

[30] Bastrom TP, Ohashi M, Bartley CE, Marks MC, Yaszay B, Lonner BS et al (2022) Factors associated with increased back pain in primary thoracic adolescent idiopathic scoliosis 10 years after surgery. Spine Deform 10(1):55–62 34251608 10.1007/s43390-021-00384-6

[31] Mimura T, Ikegami S, Oba H, Uehara M, Koseki M, Takahashi J (2019) Factors leading to postoperative pain in adolescent idiopathic scoliosis patients including sagittal alignment and lumbar disc degeneration. Eur Spine J 28(12):3085–3091 31552534 10.1007/s00586-019-06152-5

[32] Chidambaran V, Ding L, Moore DL, Spruance K, Cudilo EM, Pilipenko V et al (2017) Predicting the pain continuum after adolescent idiopathic scoliosis surgery: a prospective cohort study. Eur J Pain 21(7):1252–1265 28346762 10.1002/ejp.1025 PMC5541247

[33] Hwang SW, Pendleton C, Samdani AF, Bastrom TP, Keeny H, Lonner BS et al (2020) Preoperative SRS pain score is the primary predictor of postoperative pain after surgery for adolescent idiopathic scoliosis: an observational retrospective study of pain outcomes from a registry of 1744 patients with a mean follow-up of 3.4 years. Eur Spine J 29(4):754–760 31993788 10.1007/s00586-020-06293-y

[34] Chidambaran V, Pilipenko V, Jegga AG, Geisler K, Martin LJ (2021) Systems biology guided gene enrichment approaches improve prediction of chronic post-surgical pain after spine fusion. Front Genet 12:594250 33868360 10.3389/fgene.2021.594250 PMC8044807

[35] Chidambaran V, Mavi J, Esslinger H, Pilipenko V, Martin LJ, Zhang K et al (2015) Association of OPRM1 A118G variant with risk of morphine-induced respiratory depression following spine fusion in adolescents. Pharmacogenomics J 15(3):255–262 25266679 10.1038/tpj.2014.59 PMC4406866

[36] Ng BKW, Chau WW, Hui CN, Cheng PY, Wong CY, Wang B et al (2015) HRQoL assessment by SRS-30 for Chinese patients with surgery for Adolescent Idiopathic Scoliosis (AIS). Scoliosis 10(Suppl 2):S19 25810753 10.1186/1748-7161-10-S2-S19 PMC4331737

[37] McKean GM, Tsirikos AI (2017) Quality of life in children and adolescents undergoing spinal deformity surgery. J Back Musculoskelet Rehabil 30(2):339–346 27858696 10.3233/BMR-160558

[38] Grant DR, Schoenleber SJ, McCarthy AM, Neiss GI, Yorgova PK, Rogers KJ et al (2016) Are we prescribing our patients too much pain medication? Best predictors of narcotic usage after spinal surgery for scoliosis. J Bone Joint Surg Am 98(18):1555–1562 27655983 10.2106/JBJS.16.00101

[39] Rosenberg RE, Trzcinski S, Cohen M, Erickson M, Errico T, McLeod L (2017) The association between adjuvant pain medication use and outcomes following pediatric spinal fusion. Spine 42(10):E602–E608 27584679 10.1097/BRS.0000000000001892

[40] Segal KR, Gomez JA, Schulz JF, Alvandi LM, Fornari ED (2023) The impact of standardized recovery pathways on language barriers and inpatient pain management. Hosp Pediatr 13(11):1001–1009 37850258 10.1542/hpeds.2023-007232

[41] Yang S, Werner BC (2019) Risk factors for prolonged postoperative opioid use after spinal fusion for adolescent idiopathic scoliosis. J Pediatr Orthop 39(10):500–504 31599858 10.1097/BPO.0000000000001139

[42] Ballock RT, Seif J, Goodwin R, Lin JH, Cirillo J (2021) Clinical and economic outcomes associated with use of liposomal bupivacaine versus standard of care for management of postsurgical pain in pediatric patients undergoing spine surgery. J Health Econ Outcomes Res 8(1):29–35 33880386 10.36469/jheor.2021.21967 PMC8049745

[43] Barnett SA, Song BM, Bauer M, Nungesser ME, Leonardi C, Heffernan MJ (2023) Minimalistic approach to enhanced recovery after pediatric scoliosis surgery. Spine Deform 11(4):841–846 36935474 10.1007/s43390-023-00675-0 PMC10261149

[44] Batko I, Koscielniak-Merak B, Tomasik PJ, Kobylarz K, Wordliczek J (2020) Lidocaine as an element of multimodal analgesic therapy in major spine surgical procedures in children: a prospective, randomized, double-blind study. Pharmacol Rep 72(3):744–755 32297162 10.1007/s43440-020-00100-7 PMC7329801

[45] Koscielniak-Merak B, Batko I, Kobylarz K, Tomasik P (2018) Postoperative pain after spinal surgery in the paediatric population. Anaesthesiol Intensive Ther 50(4):252–258 30284715 10.5603/AIT.a2018.0034

[46] Changoor S, Giakas A, Sacks K, Asma A, Lang RS, Yorgova P et al (2024) The role of liposomal bupivacaine in multimodal pain management following posterior spinal fusion for adolescent idiopathic scoliosis: faster and farther with less opioids. Spine 49(2):E11–E16 37159268 10.1097/BRS.0000000000004702

[47] Fons RA, Hainsworth KR, Michlig J, Jablonski M, Czarnecki ML, Weisman SJ (2024) Perioperative methadone for posterior spinal fusion in adolescents: results from a double-blind randomized-controlled trial. Paediatr Anaesth 34(5):438–447 38288667 10.1111/pan.14843

[48] Hasan MS, Abdul Razak N, Yip HW, Lee ZY, Chan CYW, Kwan MK et al (2023) Association between intraoperative remifentanil use and postoperative hyperalgesia in adolescent idiopathic scoliosis surgery: a retrospective study. BMC Anesthesiol 23(1):177 37226107 10.1186/s12871-023-02127-8 PMC10207683

[49] Julien-Marsollier F, Assaker R, Michelet D, Camby M, Galland A, Marsac L et al (2021) Effects of opioid-reduced anesthesia during scoliosis surgery in children: a prospective observational study. Pain Manag 11(6):679–687 34102877 10.2217/pmt-2020-0100

[50] Sandhu MRS, Craft S, Reeves BC, Sayeed S, Hengartner AC, Tuason DA et al (2025) High inpatient-opioid consumption predicts extended length of hospital stay in patients undergoing spinal fusion for adolescent idiopathic scoliosis. Spine Deform 13(1):111–121 39320702 10.1007/s43390-024-00960-6

[51] Yang D, Jha S, Swallow J, Caird MS, Lopyan A, Stepanovich M et al (2022) Preoperative patient education and smaller prescription quantity reduce opioid use after posterior spinal fusion for adolescent idiopathic scoliosis: results of a prospective study. J Pediatr Orthop 42(8):e868–e873 35856498 10.1097/BPO.0000000000002215

[52] Choudhry DK, Brenn BR, Sacks K, Shah S (2019) Evaluation of gabapentin and clonidine use in children following spinal fusion surgery for idiopathic scoliosis: a retrospective review. J Pediatr Orthop 39(9):e687–e693 31503225 10.1097/BPO.0000000000000989

[53] Verriotis M, Jones L, Whitehead K, Laudiano-Dray M, Panayotidis I, Patel H et al (2018) The distribution of pain activity across the human neonatal brain is sex dependent. Neuroimage 178:69–77 29763673 10.1016/j.neuroimage.2018.05.030 PMC6062722

[54] Soffin EM, Wilson LA, Liu J, Poeran J, Memtsoudis SG (2021) Association between sex and perioperative opioid prescribing for total joint arthroplasty: a retrospective population-based study. Br J Anaesth 126(6):1217–1225 33674073 10.1016/j.bja.2020.12.046

