# Supplementary material for: Sex-specific pain management and outcomes in pediatric elective posterior spinal fusion: a database review
Source: Spine Deform. 2026 Feb 21;14(4):1165–88. doi: 10.1007/s43390-026-01306-0 (PMC13323286; doi:10.1007/s43390-026-01306-0)
Supplement: Supplementary file 1 — Supplementary file1 (PDF 122 KB) [file 43390_2026_1306_MOESM1_ESM.pdf]

Sex-Specific Pain Management and Outcomes in Pediatric Elective Posterior Spinal Fusion: A Database Review
Appendix 1. Search Strategies

Date of completed search: April 28, 2025

Database: Medline (via OVID)

Set #	Search Strategy	Results
1<br><br>Lumbar Fusion Terms	Exp Spinal Fusion/ OR ((lumbar OR lumbars OR lumbral OR spine OR spines OR spinal OR vertebrae OR vertebraes OR vertebral OR disc OR discs OR disk OR disks OR interbody OR thoracic OR thoracolumbar) adj3 (fusion OR fusions OR fused OR fusing)).ti,ab.OR (spondylodesis OR spondylosyndesis).ti,ab. OR ((lumbar OR lumbars OR lumbral OR spine OR spines OR spinal OR vertebrae OR vertebraes OR vertebral OR disc OR discs OR disk OR disks) adj2 (surgery OR surgeries OR surgical OR surgically OR operation OR operations OR operating OR operated OR operate OR operates)).ti.	55029
2<br><br>Pain Terms	Exp Pain, Postoperative/ OR exp Low Back Pain/ or exp Back Pain/ or exp Pain Threshold/ or exp Pain Management/ or exp Pain Measurement/ or exp Acute Pain/ or exp Pain, Postoperative/ or exp Pain/ or exp Pain Perception/ or exp Chronic Pain/ OR (pain OR pains OR painful).ti,ab.	1080524
3<br><br>Pediatric Terms	Exp adolescent/ OR exp child/ OR exp child, preschool/ OR exp infant OR exp infant, newborn/ OR exp Pediatrics/ OR exp Hospitals, Pediatric/ OR (adolescent OR adolescents OR adolescence OR baby OR babies OR boy OR boys OR boyhood OR child OR childhood OR children OR "emerging adult" OR "emerging adults" OR girl OR girls OR girlhood OR infant OR infants OR infancy OR juvenile OR juveniles OR kid OR kids OR minors OR newborn OR newborns OR neonatal OR neonate OR neonates OR neonatology OR neonatologist OR neonatologists OR preterm OR prematurity OR preadolescent OR preadolescents OR preadolescence OR puberty OR pubescent OR pubescence OR prepubescent OR prepubescence OR pediatric OR pediatrics OR paediatric OR paediatrics OR PICU OR Pediatrician OR pediatricians OR paediatrician OR paediatricians OR pediatric OR pediatrics OR paediatric OR paediatrics OR stepchild OR stepchildren OR schoolchild OR schoolgirl OR schoolgirls OR schoolboy OR schoolboys OR "school age" OR "school aged" OR toddler OR toddlers OR teen OR teens OR teenager OR teenagers OR teenaged OR teenage OR	5112347

	youth OR youths OR youngster OR youngsters OR "young person" OR "young persons" OR "young people").ti,ab.	
4	1 AND 2 AND 3	2354
5	Date filter: 2015 - present	967
6	4 not (case reports OR editorial OR letter OR comment OR congress).pt.	826
